# Neutron Reflectivity and Grazing Angle Diffraction

**DOI:** 10.6028/jres.098.004

**Published:** 1993

**Authors:** J. F. Ankner, C. F. Majkrzak, S. K. Satija

**Affiliations:** National Institute of Standards and Technology, Gaithersburg, MD 20899

**Keywords:** diffraction, interfaces, neutron reflectivity, polymer interfaces, surfaces, thin films

## Abstract

Over the last 10 years, neutron reflectivity has emerged as a powerful technique for the investigation of surface and interfacial phenomena in many different fields. In this paper, a short review of some of the work on neutron reflectivity and grazing-angle diffraction as well as a description of the current and planned neutron rcflectometers at NIST is presented. Specific examples of the characterization of magnetic, superconducting, and polymeric surfaces and interfaces are included.

## 1. Introduction

Total reflection of neutrons was first observed by Fermi and Zinn [1] in 1944 and was used to determine the neutron scattering lengths of various elements. Since then, the neutron’s optical properties have been used in a variety of studies in physics, chemistry, metallurgy, and biology. Over the last ten years, neutron reflectivity has emerged as a powerful technique for the investigation of surface and interfacial phenomena in many different fields. Although similar studies on surfaces can be performed with x-ray reflectivity, neutrons provide a unique advantage because isotopic substitution can be used to achieve large contrasts in the scattering density. Most notably, the isotopic labeling of hydrogen and deuterium has been used extensively to study polymer thin films [2]. Neutrons also couple to atomic magnetic moments and hence specular reflection of polarized neutrons is a sensitive probe of surface magnetic phenomena [3].

In this paper, we will present a short review of some of the work on neutron reflectivity and grazing-angle diffraction as well as a description of the current and planned neutron rcflectometers at NIST. Specific examples of the characterization of magnetic, superconducting, and polymeric surfaces and interfaces will be included. This will not be an exhaustive review of the field —for some recent reviews on the subject, see Refs. [2] and [4].

## 2. Basic Principles

**Fig. 1:**
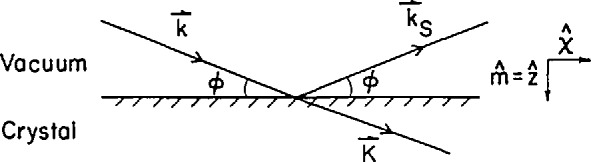
Diffraction geometry for specular reflectivity. The incident beam *k* strikes the surface at angle *ϕ* and the reflected beam *k*_s_ exits at the same angle.

In neutron reflectivity experiments an incident beam of neutrons strikes the surface of a flat sample at an angle *ϕ* (Fig. 1). Neutrons striking the surface undergo refraction and reflection if the refractive indices on the opposite sides of the interface are different. The refractive index for neutrons can be defined as

$$\begin{array}{l} n_{1}=\frac{k_{1}}{k_{0}}\sqrt{1-\frac{V_{1}}{E}}\\ =\sqrt{1-\frac{4\pi N_{1}\left(b_{1}\pm p_{1}\right)}{k_{0}^{2}}}, \end{array}$$ (1)

where *k*_1_ and *k*_0_ are the neutron wavevectors in a medium corresponding to a potential energy *V*_1_ and in the vacuum, respectively; *E* is the vacuum neutron kinetic energy, *N*_1_ the number of atoms per unit volume, and *b*_1_ and *p*_1_ the average nuclear and magnetic scattering lengths. The magnetic term describes the special case of a ferromagnetic medium so we see that a ferromagnetic material is birefringent, with the scattering length being proportional to the magnitude of the average ordered atomic magnetic moment. In the case where absorption in nonnegligible, *b*_1_ is a complex quantity. With a few exceptions, the nuclear scattering length *b*_1_ for neutrons is a positive quantity, meaning that the refractive index is less than one. Therefore, below a critical angle given by

$$\phi_{1c}={\left(\frac{4\pi N_{1}b_{1}}{k_{0}^{2}}\right)}^{1/2},$$ (2)

the neutrons are totally reflected and only an exponentially damped evanescent wave penetrates into the sample. For typical materials, *n*_1_ is such that the critical angle for total external reflection is on the order of (1°/nm)λ, where λ is the de Broglie wavelength of the incident neutrons. Beyond the critical angle, the reflectivity decreases rapidly.

For specular reflection of neutrons the angle of reflection is the same as the angle of incidence. The wavevector corresponding to the specular scattering process is *Q* = |*k*_0s_ − *k*_0_| = 2*k*_0_ sin *ϕ*, where *k*_0_ and *k*_0s_ are the incident and specular wavevectors. The reflectivity measured as a function of *Q* is related to the neutron refractive index profile normal to the surface. A number of different methods exist for calculating the reflectivity from a given scattering density (*Nb*) profile.

Since the momentum transfer in specular reflectivity is only in the direction normal to the surface, this scattering can be described in terms of the solution of the one-dimensional wave equation (derived from the Schrödinger equation),

$$\psi \prime \prime \left(z\right)+\left(\frac{Q^{2}}{4}-4\pi N_{1}b_{1}\right)\psi \left(z\right)=0,$$ (3)

where $\psi \left(z\right)=e^{ik_{0z}}z)$ is the neutron wave function and $\hat{z}$ is the unit normal to the surface. For a stratified medium where *b* and/or *N* are functions of the depth z, the reflectivity can be calculated by imposing continuity on the wave function and its first derivative sequentially at each boundary between layers of constant potential in piece-wise continuous fashion using well-known matrix methods. Then, by modelling the refractive index profile as a function of depth z, an attempt to fit an observed reflectivity profile can be made. The reflectivity can also be calculated from the refractive index profile using methods developed in classical optics [5]. These methods are completely equivalent.

In the kinematical limit (when the reflectivity is much less than 1), one can write this reflectivity as

$$R\left(Q\right)={\left|r\left(Q\right)\right|}^{2}\approx {\left|\frac{4\pi Nb}{Q}\int_{-\infty }^{\infty }\rho \left(z\right)e^{iQz}dz\right|}^{2},$$ (4)

where *Nb* is the average scattering density of the sample and *ρ*(*z*) is the deviation from that average density as a function of depth z. Integrating this expression by parts, we can then express the reflectivity as

$$R\left(Q\right)\approx \frac{{\left(4\pi Nb\right)}^{2}}{Q^{4}}{\left|\int_{-\infty }^{\infty }\frac{\partial \rho \left(z\right)}{\partial z}\;e^{iQz}dz\right|}^{2}.$$ (5)

This expression shows that scattering density gradients determine the specular reflectivity. Hence, the reflectivity will be most sensitive to those portions of the density profile with the largest gradients. For a more detailed general discussion on reflectivity, see Refs. [6–8] (for polarized beams, see Refs. [9, 10]).

## 3. Instruments

Two basic methods of measuring neutron reflectivity have been developed. A wide range of wavevectors *Q* can be achieved by sending a broad-spectrum beam onto a sample at a fixed angle of incidence. Time-of-flight (TOF) is used to measure the wavelengths of the reflected neutrons. The TOF method has been utilized very successfully at pulsed neutron sources, originally by G. P. Felcher at Argonne National Laboratory. In addition to the pioneering instrument at Argonne, TOF reflectometers have been built at several different laboratories around the world, including the ISIS pulsed source at Rutherford Lab in the U.K. [11], the Los Alamos National Lab pulsed source [12], and the instrument at the Orphee reactor at Saclay [13]. The TOF method possesses the advantage of constant sample illumination for all wavevectors. The relative resolution *δQ*/*Q* is dominated by the angular divergence of the beam *δθ* and is, therefore, constant over the reflectivity profile. However, the actual resolution *δQ* varies widely over the whole *Q* range. For a review of TOF instruments, see Refs. [9] and [11].

**Fig. 2:**
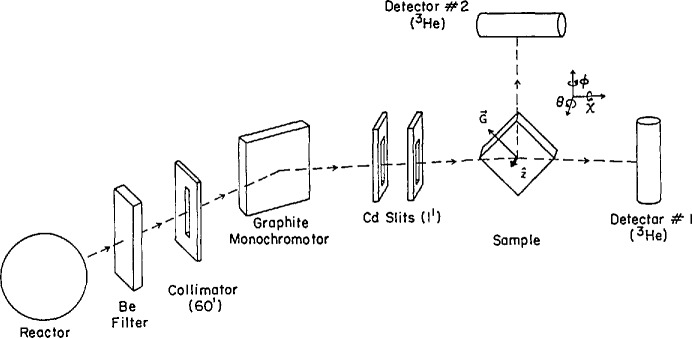
Schematic of neutron reflectometer BT-7 at NIST. The specularly reflected beam enters detector 1. Detector 2 is used for grazing-angle diffraction.

On a reactor-based reflectometer, one has the option of using a monochromatic beam and a conventional *θ*−2*θ* scan for varying *Q.* In this method the illuminated area of the sample varies with angle, which requires that samples be large enough to intercept a substantial fraction of the beam at low angles. A general assumption in the field has been that long-wavelength neutrons are required for a reactor-based, fixed-wavelength reflectometer. However, we have shown that 0.235 nm neutrons can be used very effectively to measure profiles down to the range of 2–3 × 10^−7^ absolute reflectivity. A schematic of the BT-7 reflectometer at NIST is shown in Fig. 2. A filtered and collimated monochromatic (0.235 nm) beam of neutrons is incident on the sample. The collimation of the slits before the sample is continuously variable. In a typical scan, the slit just before the sample varies from 50 μm to about 1 mm with increasing *Q*, yielding angular divergences in the range of 0.3–1 mrad. The wavelength spread is mainly determined by the collimation before the monochromator and is *δ*λ/λ∼0.01. The *Q* resolution at small momentum transfers (*Q* ≈0.2 nm^−1^) is about 0.02 nm^−1^ and at large transfers (Q ≈2 nm^−1^) is of order 0.05 nm^−1^. A resolution of 0.02 nm^−1^ means that one can resolve Kiessig fringes from a film 200–300 nm thick. Reflectivities down to the 2–3 × 10^−7^ can be measured on this instrument. The present arrangement requires samples to be in the vertical geometry. It is also worth pointing out that this instrument can be used in polarized-beam mode, by means of polarizing supermirrors. Both incident and exit polarization analyses are available.

**Fig. 3:**
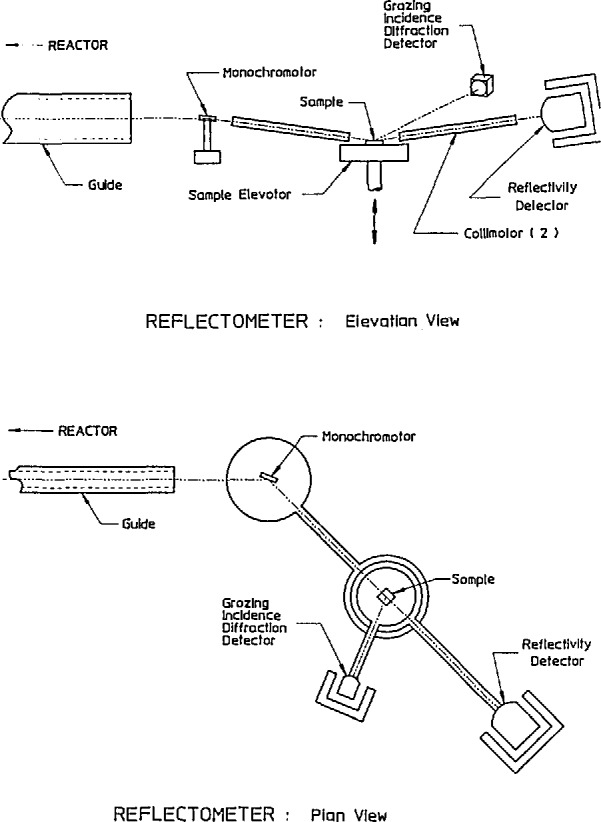
Elevation and plan views of the cold neutron reflectometer under eonstruetion at NIST.

Another reflectometer to be located in the NIST guide hall is presently under construction. This instrument will allow the sample to be in the horizontal position, thereby facilitating the study of liquid-vapor interfaces. Figure 3 shows both the elevation and plan views of the reflectometer. A neutron beam from the guide tube is monochromated and deflected down onto the sample by a graphite crystal. The angle of incidence is changed by raising or lowering the sample table and changing the tilt of the monochromator. Two detectors will be available—one for reflectivity and the other for grazing-angle diffraction, either or both of which will be position-sensitive detectors. Four different wavelengths, from 0.235 to 0.55 nm will be available. This instrument will be able to measure reflectivities in the 10^−7^ range.

Other reactor-based fixed-wavelength reflec-tometers around the world include ones at HFBR at Brookhaven National Lab, Jülich in Germany, and a new reflectometer/grazing-angle diffractometer at the Institut Laue-Langevin in Grenoble. We will now review some of the scientific studies being presently done or reported in the literature using neutron reflectometers. Parts of this review have already appeared in a recent paper by Majkrzak and Felcher [15].

### 3.1 Superconducting and Magnetic Layers

The interaction between the neutron’s dipole moment ***μ*** and an atomic magnetic moment as characterized above by the scattering length p can also be expressed in a more general form as the potential energy *V*= **− *μ·B*** where ***B*** is the local magnetic induction. One of the first magnetic profiles examined by neutron reflection was that of the penetration of an externally applied magnetic field into a superconducting material [16]. In superconducting materials the Meissner effect requires that *B* = 0 even in the presence of an external field (below a critical value). The shielding is provided by the onset of supercurrents in proximity to the surface. The penetration depth of the magnetic field is inversely proportional to the ability of the material to magnetically shield itself. Using a polarized neutron beam, spin-dependent reflectivity profiles can be measured because of the diamagnetic response of the superconductor to the applied magnetic field. The superconducting penetration depth in niobium, for example, has been directly measured by neutron reflectivity [16] to be 41 ±4 nm, in reasonable agreement with theory.

In ferromagnetic materials, the magnetic contribution to the reflectivity can be much stronger than that observed in the case of a superconductor. It was predicted [17] and subsequently demonstrated [18] that a magnetic monolayer could be detected by neutron reflectivity. The effects of reduced dimensionality on interfacial magnetic states and corresponding critical behavior are of considerable current interest [19].

### 3.2 Magnetic Multilayers

**Fig. 4:**
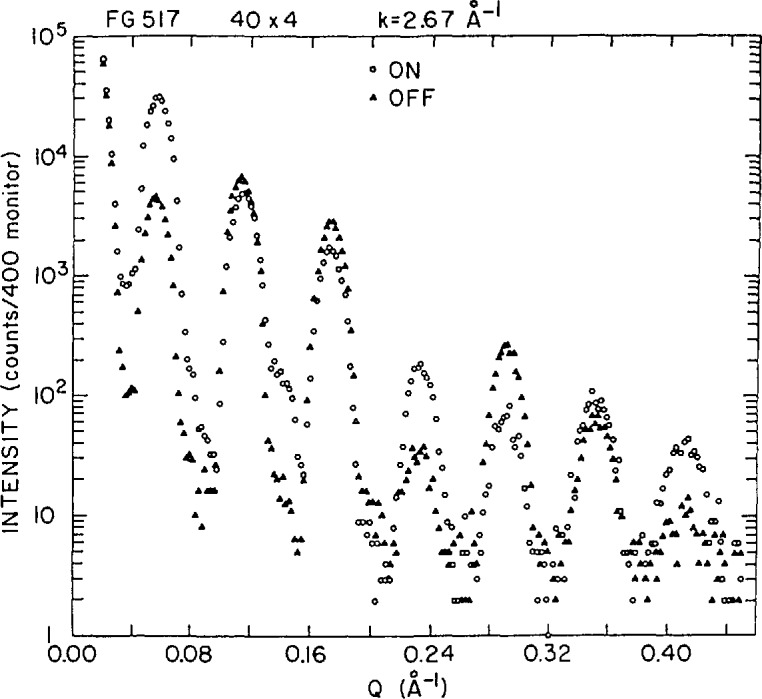
Polarized neutron diffraetion sean with *Q* perpendicular to the film planes of an Fe-Ge multilayer. Seven multilayer peaks with periodicity *Q* = 2*πm*/*D* are shown beginning with *m*=1. The “ON” (“OFF”) data points correspond to incident neutrons in the “ + ” (“ − ”) spin eigenstate (after Ref. [22]).

By depositing a coherent superposition of a number of identical bilayers, the neutron reflectivity can be appreciably enhanced. Early neutron reflectivity work on magnetic multilayers has been reviewed by Endoh [20, 21]. However, sample quality, particularly the failure to maintain a regular bilayer period (which results in a relatively rapid broadening of higher-order reflections), limited initial studies in the detail of the magnetization profile which could be obtained perpendicular to the plane of the film. If the bilayer period is well-defined, then a sufficient number of harmonics may be observed to extract a magnetization profile with a resolution of several tenths of a nanometer. In Fig. 4 is shown polarized neutron reflectivity data for an Fe-Ge multilayer [22] with a bilayer spacing *D* of approximately 10.8 nm. Ignoring slight refraction corrections, multilayer reflections occur at integer multiples of 2*π/D.* Note that the ratio of the two reflectivities corresponding to the “ + ” and “ − ” neutron spin eigenstates is not the same for all harmonics. This immediately implies that the magnetization profile across the ferromagnetic layer is nonuniform. X-ray and neutron diffraction measurements performed at higher values of *Q* about the Fe(110) peak position have revealed that the central sections of the Fe layers are composed of microcrystallites which have a strong preferred orientation of their close-packed (110) planes parallel to the substrate plane and which are at the same time randomly rotated about the growth direction (the Ge layers were found to be amorphous). Further quantitative analysis of the reflectivity measurements yielded a magnetization profile with an interfacial region in which the moment was markedly reduced due primarily to inter-diffusion.

It is important to note that although it may be possible to obtain an accurate magnetization profile along the growth direction in a given multilayer structure from the neutron reflectivity data, the correct interpretation of this profile requires proper consideration of not only interdiffusion but of interfacial roughness and/or waviness. To distinguish diffusion from roughness, diffraction scans orthogonal to the longitudinal specular reflectivity scans must be done so that a component of ***Q*** lies in the plane of the interface. The nonspecular scattering also gives information about possible multiple or simultaneous scattering effects and in-plane composition modulation. An effort is currently being made to extend the methods applied to the treatment of roughness for a single surface [23] to multiple interfaces.

It is also of some interest to point out that ferromagnetic thin film multi-bilayers can be used as efficient neutron polarizers by properly matching *Nb* and *Np* (see, for example Refs. [24] and [25] and references therein).

### 3.3 Polymer Interfaces

As mentioned in the introduction, neutron scattering amplitudes are isotope dependent. For hydrogen and deuterium the difference in amplitudes is relatively large so that by selective deuteration the scattering density contrast between different polymers or a polymer and solvent, for example, can be greatly enhanced. Neutron reflectivity measurements which yield the refractive index profile normal to the surface have been performed on numerous organic film structures in order to study interdiffusion and mixing as well as the adsorption of surfactants, polymers, and fatty-acids (see Refs. [26–28] and references therein). Langmuir-Blodgett film systems have also been studied by this technique [29–32].

**Fig. 5:**
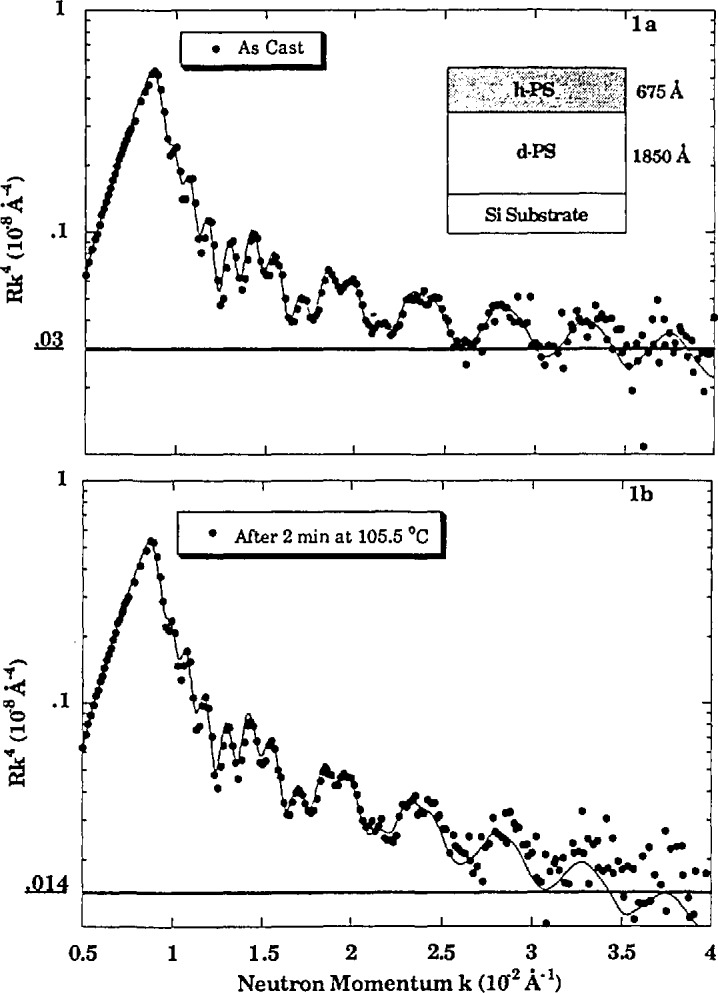
Neutron reflectivity profile of a bilayer of polystyrene in which one of the layers has been deuterated. The data were collected before and after annealing. The solid lines represent the reflectivities calculated for the layer thicknesses given in the inset. After annealing, the reflectivity decreases as a result of interdiffusion.

As a specific example, neutron reflectivity can be used to monitor the early stages of interdiffusion of a bilayer heated to a suitable temperature, either taking reflectivity scans short in comparison with the diffusion time or quenching the sample after each anneal. In Fig. 5 is plotted the reflectivity $\left(\times k_{0z}^{4}\right)$ of a bilayer of polystyrene of 230,000 molecular weight as cast and after a short time anneal. The contrast between the two layers is obtained by deuterating the bottom layer. The solid lines between data points represent the reflectivities calculated for the layer thicknesses presented in the insert. After the anneal the reflectivity decreases as a result of interdiffusion over a thickness 〈z^2^〉^1/2^ = 3.0 nm.

**Fig. 6:**
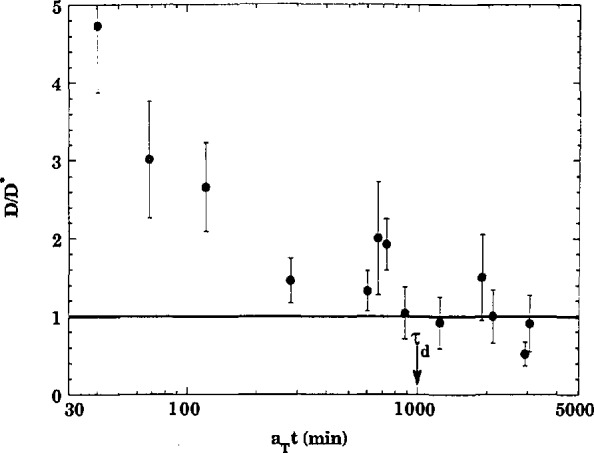
Results of neutron reflectivity measurements showing that the diffusion constant for polymer systems becomes constant after a molecule has moved to a full radius of gyration.

The interdiffusion of polymers is significantly different from that of simple molecules, where the latter may be represented as unstructured hard balls whose effective motion is completely described by the center of mass. The polymers have instead a characteristic size (radius of gyration) and each molecule is deeply entangled with its neighbors. These entanglements constrain their motion. The segments between the entanglements are relatively free to move, but this does not result in any motion of the center of mass of the molecule. The molecule as a whole may move only by sliding between entanglements with a repetitive motion. According to a theoretical model of polymer diffusion, the diffusion constant *D* of the molecule (which in conventional diffusion theory is given by 〈*z^2^*〉 = 2*Dt*) should decrease significantly from the time in which only segmental motion has taken place to the time in which the molecule has moved a full radius of gyration (reptation time) after which the diffusion coefficient should become constant. Figure 6 shows that the neutron reflection experiments are consistent with this picture.

**Fig. 7:**
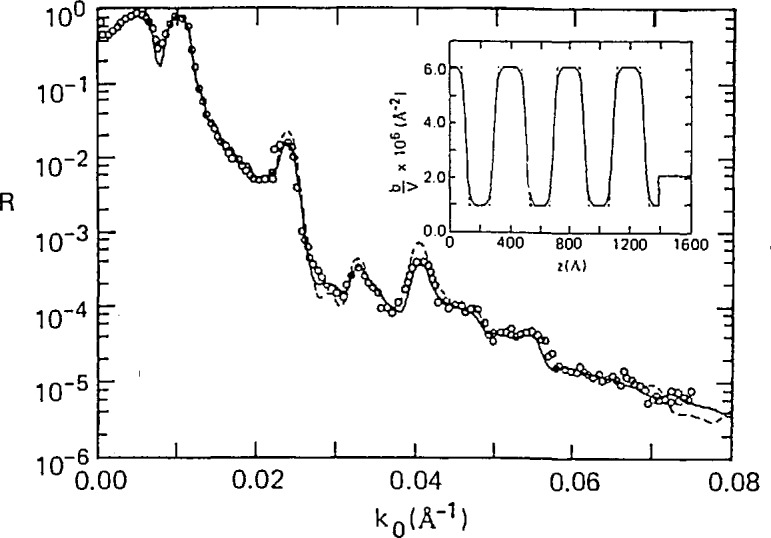
Comparison of the experimentally measured neutron reflectivity profile and those calculated using a linear (⋯) and hyperbolic tangent (−) function to describe the segment density profile at the interface between the PS and PMMA microdomains for P(d-S-b-MMA) 100 K (after Ref. [37]).

As another illustration of what can be learned from neutron reflectivity measurements, consider the surface induced ordering of diblock copolymer films. Block copolymers of polystyrene (PS) and polymethylmethacrylate (PMMA) are used as surfactants, compatibilizing agents, and adhesives in biomedical and microelectronics applications. The morphology of these copolymers near surfaces can be significantly affected by the difference in the surface free energy of the two blocks and their affinity for the substrate. Depending on the interaction between the two blocks, the copolymer can be either homogeneously mixed or separated into lamellar microdomains which in the bulk are randomly oriented. However, in properly annealed films, these lamellar microdomains orient parallel to the free surface [33, 34] which is an ideal configuration for determining the compositional profile across the interfacial regions. Other methods such as transmission electron microscopy are limited by the feasibility of staining procedures and/or spatial resolution. These limitations do not apply to neutrons—the reflectivity study performed by Anastasiadis et al. [35] clearly showed that not only does a PS layer preferentially locate at the air-copolymer interface and PMMA at the substrate, but the layer thicknesses at the air and substrate interfaces are half those found in the bulk. Furthermore, an interfacial region was observed and determined to have a width of 5.4 nm to an accuracy of about 0.2 nm. In addition, evidence of surface-induced ordering of these copolymers in the phase-mixed state (above the bulk microphase separation temperature) was found, characterized by an exponentially damped, oscillatory density profile normal to the film surface as recently predicted by mean-field theory [36]. Further neutron reflectivity work on this particular system [37] has demonstrated that the measured reflectivity is also sensitive to the shape of the density profile across the interfacial region as shown in Fig. 7.

### 3.4 Reflection at Solid-Solution Interfaces

A distinct advantage in using neutrons for reflectivity measurements is their ability to traverse macroscopic distances in single crystals such as silicon, quartz, or sapphire with relatively little loss. This makes it possible to use one of these crystals not only as a substrate on which a thin film or superlattice can be grown, but also as the incident medium. That is, the neutron beam can enter into one side of the crystal at nearly normal incidence and subsequently reflect at a glancing angle from the crystal-film interface. The opposite side of the film can then be placed in intimate contact with a liquid solution. A similar idea was first used [38] to measure the scattering length of liquid ^3^He.

**Fig. 8:**
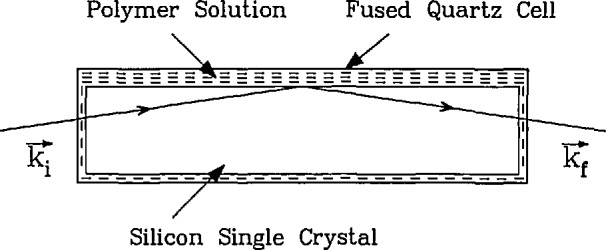
Schematic (top view) of the cell used for neutron reflectivity measurements at solid-liquid interfaces. A large silicon single crystal is enclosed in a cell made of fused quartz with two 0.5 mm-thick windows for the incident and reflected neutron beams. The volume surrounding the Si crystal contains the polymer and solvent. The most important feature of this cell is that the incident and reflected beams pass through the bulk silicon crystal.

**Fig. 9:**
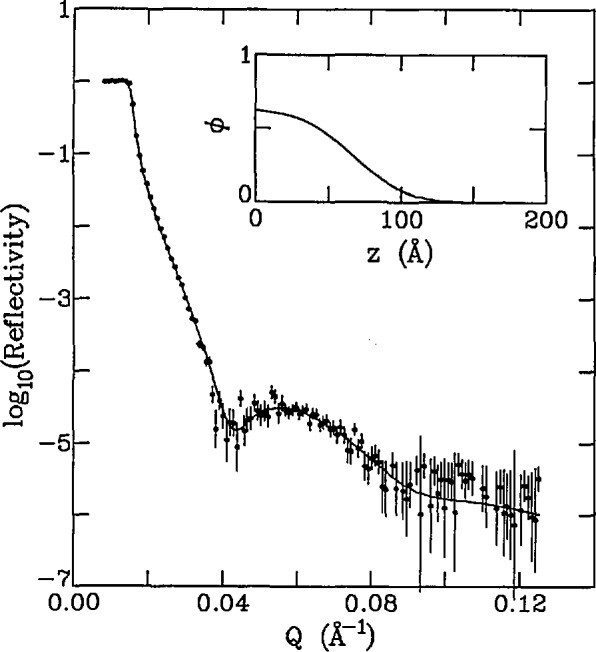
Open circles show the reflectivity from a silicon surface immersed in pure deuterated cyclohexane (DCH); closed circles show reflectivity from same surface in solution of end-carboxy-latcd polystyrene (PS-COOH) in DCH.

A schematic of such a cell, which was recently used to study polymers adsorbed from solution, is shown in Fig. 8. A recent example of the study of polymer brushes at the Si/solution interface [39] is shown in Fig. 9. The polymer used in this case was end-group carboxylated polystyrene (hydrogenated) with deuterated cyclohexane as the solvent. The carboxylated end group of the polymer is strongly adsorbed onto the silicon surface. A well-defined minimum in the reflectivity profile is characteristic of an adsorbed polymer film at the silicon-solution interface. The inset of Fig. 9 shows the concentration profile of this polymer brush at the interface determined by a nonlinear least squares fit of the reflectivity data.

The liquid used in such a cell can even be an aqueous solution which is part of an active electrochemical cell. For example, the diffusion of either hydrogen or deuterium into a metal host film or superlattice can be measured [40] since H and D have scattering lengths which are comparable to those of the much heavier metal atoms. Although the density of hydrogen in thin films or multilayers of metal hydrides can be inferred from an expansion of the host lattice by conventional x-ray diffraction techniques, neutron reflectivity measurements yield the hydrogen density profile directly. By combining neutron reflectivity with higher angle x-ray and neutron diffraction, it is possible in principle to determine the absolute amount of hydrogen soluble in a given metal layer and whether the hydrogen occupies only interstitial sites in the host lattice or resides partly in voids.

### 3.5 Epitaxial Superlattices

Advances in molecular beam epitaxy techniques have resulted in the growth of single crystal, magnetic rare earth and transition metal superlattices with well defined periodicities (see, for example, Ref. [19] and references therein). Multilayers such as these are ideal for studying the effects of finite size on the magnetic behavior as discussed earlier. However, in the case of the rare earths the indirect exchange coupling responsible for the magnetic ordering is mediated by the conduction electrons so that the artificially imposed compositional modulation might in itself be expected to perturb the magnetic state of the superlattice. This is indeed found to occur in a number of superlat-tices composed of a magnetic rare earth alternating with the nonmagnetic metal yttrium.

**Fig. 10:**
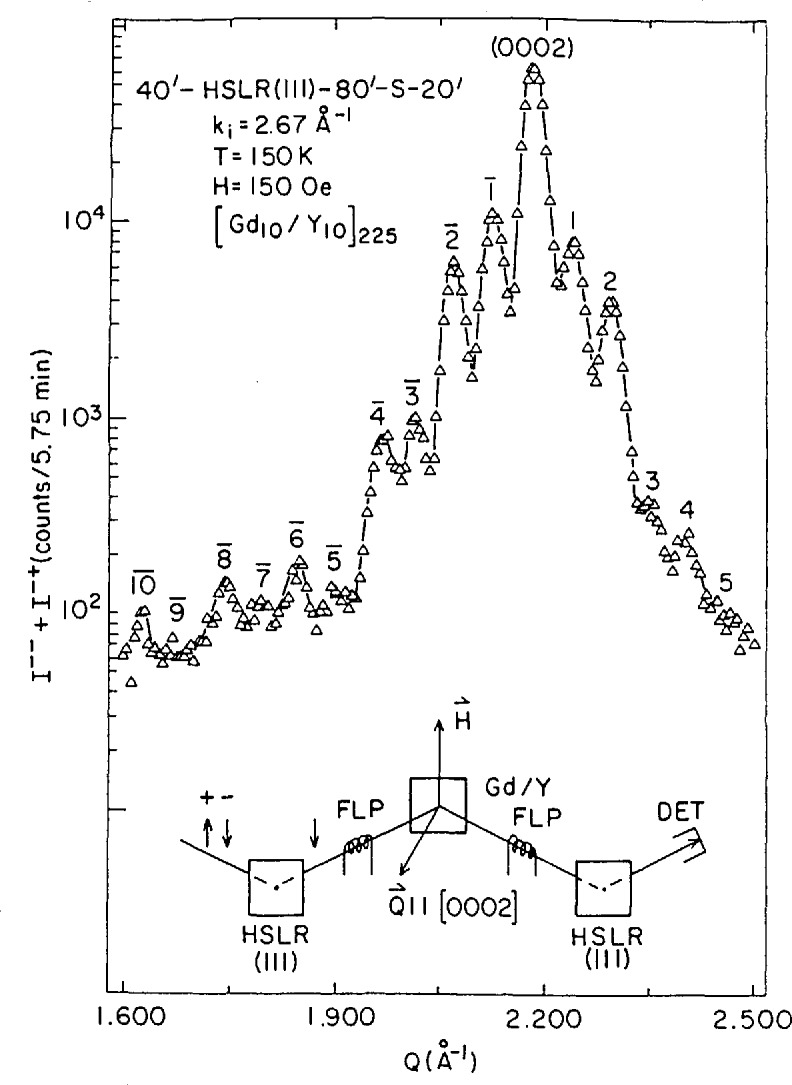
Polarized neutron diffraction data for a Gd-Y supcrlatticc in which the ferromagnetic Gd slabs or layers are antifcrromagnctically coupled across the intervening nonmagnetic Y. The odd-integer superlattiec satellite peaks occur at positions which correspond to a doubled chemical superlattice unit cell and arc the result of spin-flip scattering only. The lower part of the figure is a schematic of the instrumental configuration where HSLR(111) denotes the reflecting planes of the Cu_2_MnAI Heusler polarizing crystals (after Ref. [42]).

Polarized neutron diffraction studies have shown that in Gd-Y superlattices, for certain Y layer thicknesses, the ferromagnetic Gd layers (bulk Gd is a simple ferromagnet) align antiparallel to one another in an antiphase domain structure [41]. The odd integer, SF satellites shown in Fig. 10 occur at positions corresponding to a doubled superlattice spacing and are characteristic of the antiparallel Gd layer alignment. An oscillatory dependence of the Gd layer moment configuration on the intervening Y layer thickness has been interpreted to be a consequence of the coherent propagation of magnetic correlations across the Y via the Ruderman-Kittel-Kasuya-Yosida (RKKY) interaction.

Neutron diffraction studies have also shown that long range magnetic order in the form of spirals and noncollinear structures develops in other rare earth superlattice systems including Dy-Y, Ho-Y, Er-Y, and Gd-Dy (for reviews of this work see Refs. [42] and [43]). In addition to the effects of the imposed composition modulation and finite layer thickness, the epitaxial strain due to lattice mismatch at the interface between two dissimilar elements can, through the magnetoelastic energy, play an important role in determining what magnetic order develops [44].

## 4. Grazing Angle Diffraction

There are a number of methods for studying surface and interfacial phenomena with neutrons and X rays that involve diffraction from ordered crystalline structures (as opposed to the purely refractive effects used in specular reflectivity experiments). Zeilinger and Beatty [45] carried out measurements using asymmetric scattering geometries that yield information about the surface structures of perfect crystals. Al Usta, Dosch, and Peisl [46] have investigated the possibility of measuring the diffuse tails of bulk Bragg peaks (“truncation-rod scattering”) with neutrons but find also that low intensity limits the applicability of the method to nearly perfect crystals. The most promising application of crystalline diffraction to surface neutron studies is the grazing-angle diffraction geometry because it possesses the potential to observe imperfect crystals.

**Fig. 11:**
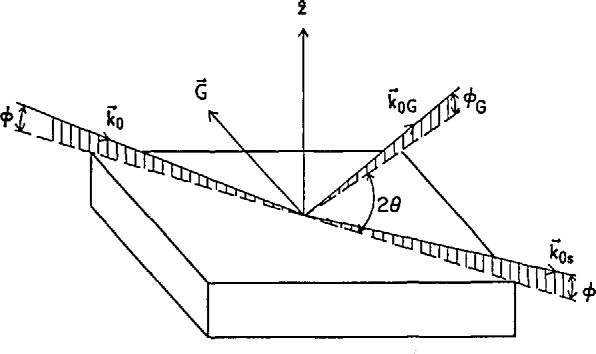
Grazing-angle diffraction geometry. Simultaneously with the specular reflectivity process (*k*_0s_), a sample is oriented such that the components of *k*_0_ and *k*_0*G*_ in the surface plane satisfy the Bragg condition with reciprocal lattice vector ***G***.

Grazing-angle diffraction is an application of specular reflectivity that allows one to study the in-plane structure of near-surface and interfacial layers. The theory of this diffraction geometry, the distorted-wave approximation, has been worked out in a number of papers [47–49]. For grazing-angle diffraction, one uses the amplitude of the evanescent transmitted wave of the specular reflection process as the illuminating field for Bragg diffraction. In Fig. 11, the incident beam *k*_0_ strikes the sample surface near the total reflectivity threshold and excites a specularly reflected beam *k*_0s_, with the sample being oriented such that a reciprocal lattice vector ***G*** satisfies the Bragg condition with the components of wavevectors *k*_0_ and *k*_0G_ in the plane of the sample surface: ***Q***_‖_ = (*k*_0G_ −*k*_0_)_‖_ *= **G**.* The out-of-plane components control the depth of illumination,

$$Q_{1z}=\sqrt{k_{0z}^{2}-4\pi N_{1}b_{1}}+\sqrt{k_{0Gz}^{2}-4\pi N_{1}b_{1}},$$ (6)

where *N*_1_ is the atomic density of the scatterers in the crystal and *b*_1_ their average scattering length. The characteristic depth probed is simply given by the inverse of the imaginary part of *Q*_1z_ and typically is of order 5–10 nm at the lowest angles of incidence and exit. Taking into account these refractive corrections, one proceeds with the calculation of the diffracted intensity according to conventional kinematical theory (50). The resulting grazing-angle diffracted intensity is

$$\begin{array}{l} I\left(Q\right)=\\ I_{0}{\left|t_{1}\left(k_{0z}\right)\right|}^{2}{\left|t_{1}\left(k_{0Gz}\right)\right|}^{2}{\left|F_{1}\left(Q_{\left|\right|}\right)\right|}^{2}\left|\frac{e^{iQ_{1z}M_{1}a_{1}}-1}{e^{iQ_{1z}a_{1}}-1}\right|\delta \left(Q_{\left|\right|}-G\right), \end{array}$$ (7)

where the exponential terms result from the summation over crystal planes normal to the surface, with *M*_1_ being the number of atomic layers and *a*_1_ their lattice spacing. The delta function expresses the Bragg condition with the in-plane wavevector components and *F*_1_ is the structure factor for the reflection. The terms *t* are simply the Fresnel transmitted amplitudes of the incident and diffracted waves,

$$t_{1}\left(k\right)=\frac{2k}{k+{\left[k^{2}-4\pi N_{1}b_{1}\right]}^{1/2}}.$$ (8)

Just as is the case for reflectivity, the above formulae can be derived for an arbitrary number of diffracting layers [51, 52].

**Fig. 12:**
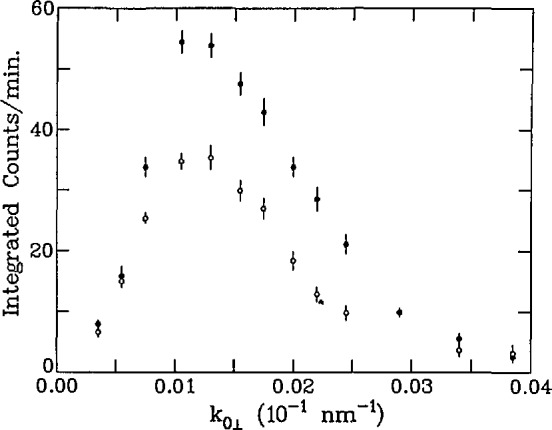
Polarized neutron grazing-angle diffraction from $\left(11\bar{2}0\right)$ reflection of an Y(0001)/Gd(0001) film. The solid circles were measured with neutrons polarized parallel to the sample magnetization and the open eireles anti-parallel. The points represent values for fixed incident *k*_0z_, integrated over a broad range in *k*_0Gz_ and over a 2.2 cire width in diffraction angle *θ.*

The primary obstacle to the development and application of grazing-angle neutron diffraction techniques is the relatively low flux of even the most powerful research nuclear reactors currently in existence (compared with x-ray synchrotron sources). Consequently, the first attempts to implement this diffraction scheme included various compromises intended to increase the neutron count rate at the detector [14, 53]. The easiest way to increase the count rate is to relax the collimations of the Bragg angle *θ* and the diffracted-beam grazing angle *ϕ_G_* (see Fig. 11). Relaxation of the Bragg angle collimation (*δθ* ∼ 2°) degrades the in-plane resolution of the instrument, but presents no problem for isolated Bragg peaks, while Eq. (6) shows that as long as one provides good collimation on the incident beam (*δϕ*∼0.015°), one can relax the diffracted-beam collimation (*δϕ_g_*∼ 1.5*°*) and still retain some depth sensitivity. Figure 12 shows a plot of the first polarized-beam grazing-angle diffraction measurements [51], which were performed using this scheme. The intensity of the reflected-diffracted beam is plotted vs. the incident wavevector component normal to the surface (*k*_0z_ = 2πsinϕ/λ) for the $\left(11\bar{2}0\right)$ in-plane Bragg reflection of an epitaxially grown film consisting of 15 nm Y(0001) on Gd(0001) measured at *T* = 150 K. As the incident angle increases, the neutrons penetrate through the magnetically dead Y layer into the ferromagnetically ordered Gd which, because of this ordering, has a different structure factor and penetration depth dependence for spin-up and spin-down neutrons. This sensitivity to magnetic order allows one in principle to determine the depth dependence of the magnetization in the sample.

There are a number of improvements which can and have been made to the instrumentation and in the data analysis that should in the near future establish the grazing-angle neutron technique as an important probe in interface science. A1 Usta, Dosch, Lied, and Peisl [54] have demonstrated the importance of using a position-sensitive detector to record the data as a function of exit diffraction angle *ϕ_G_.* This angular differentiation is necessary for two reasons: first, one would like to have more precise control of the scattering depth and, second, since the neutron absorption cross section is negligible for most materials there exist a number of spurious refracted beams coming off the sample which must be taken into account before one can see the true surface scattering (this effect is fortuitously not as large in the polarized-beam measurement described above, due to the large absorption cross section of Gd). They have demonstrated that one can obtain adequate intensity with this arrangement, albeit at the cost of greater effort expended in alignment. With the implementation of the appropriate hardware for these measurements comes the need for an adequate formalism to treat the data. The authors have described a formalism for a multi-layer treatment of grazing-angle diffraction [51,52] which is quite similar to the methods used in reflectivity analysis and plan to discuss both reflectivity and grazing-angle modeling in an upcoming paper. With these tools in place, grazingangle diffraction is poised to become an important new probe of magnetic and hydrogen-bearing materials, substances to which neutrons are uniquely sensitive.

## 5. Summary

The effectively simple description of the scattering of neutrons by condensed matter and the neutron’s sensitivity to magnetic moments and light isotopes, particularly hydrogen and deuterium, often outweigh the neutron’s relatively weak interaction strength and limited source intensities in probing surfaces and interfaces. There exist numerous examples, several of which have been given in this brief article, of how neutron reflectivity, superlattice diffraction, and grazing angle diffraction methods can be applied to study surface and interfacial phenomena in thin films of magnetic materials, polymers, superconductors, metal hydrides, and electrodes. As thin film and multilayer preparation techniques progress, neutron scattering studies should continue to play an important role in the characterization of the properties of these novel synthetic materials.
